# Supplementary figures and images for: Developing bottom drifters to better understand the stranding locations of cold-stunned sea turtles in Cape Cod Bay, Massachusetts
Source: PeerJ. 2023 Aug 30;11:e15866. doi: 10.7717/peerj.15866 (PMC10474834; doi:10.7717/peerj.15866)

a

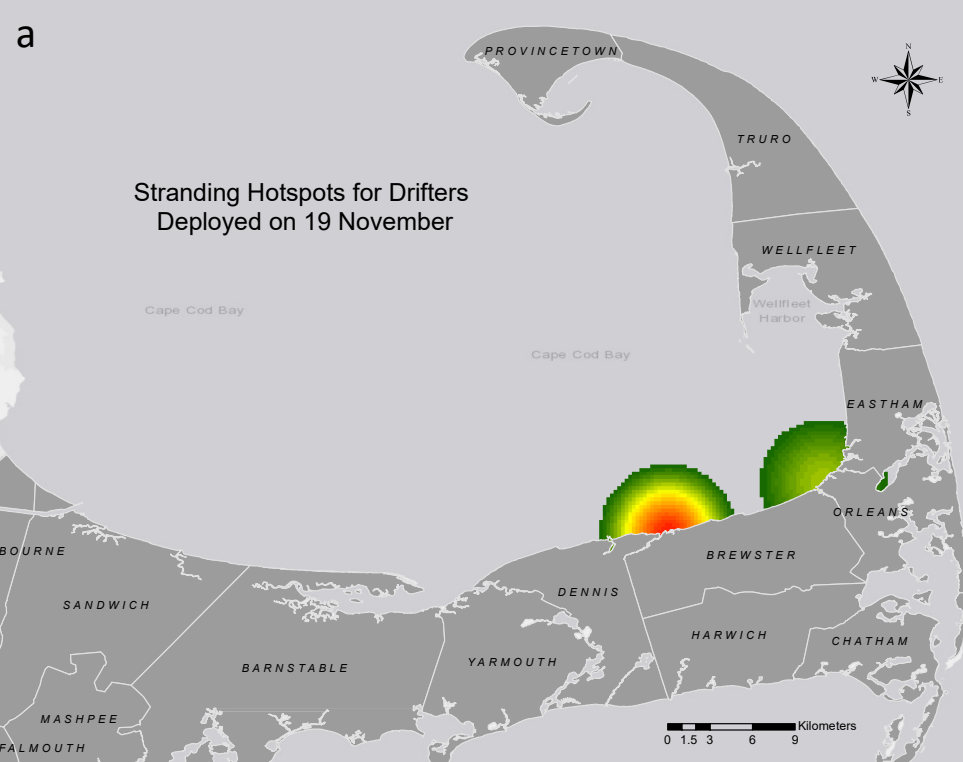

b

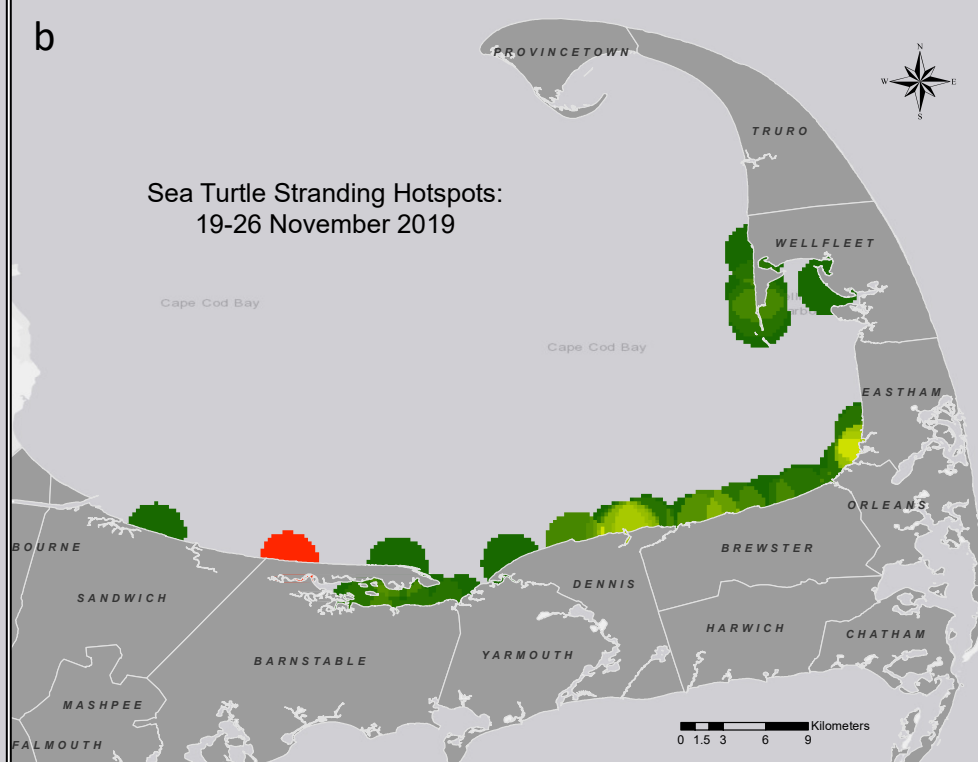

Supplement: Supplemental Information 1 — (a) Drifters (n = 6) deployed on 19 November. (b) Cold-stunned sea turtle strandings (n = 66) from 19–26 November. Red indicates the highest number of data points, while yellow indicates intermediate and green indicates the lowest number. [file peerj-11-15866-s001.pdf]

a

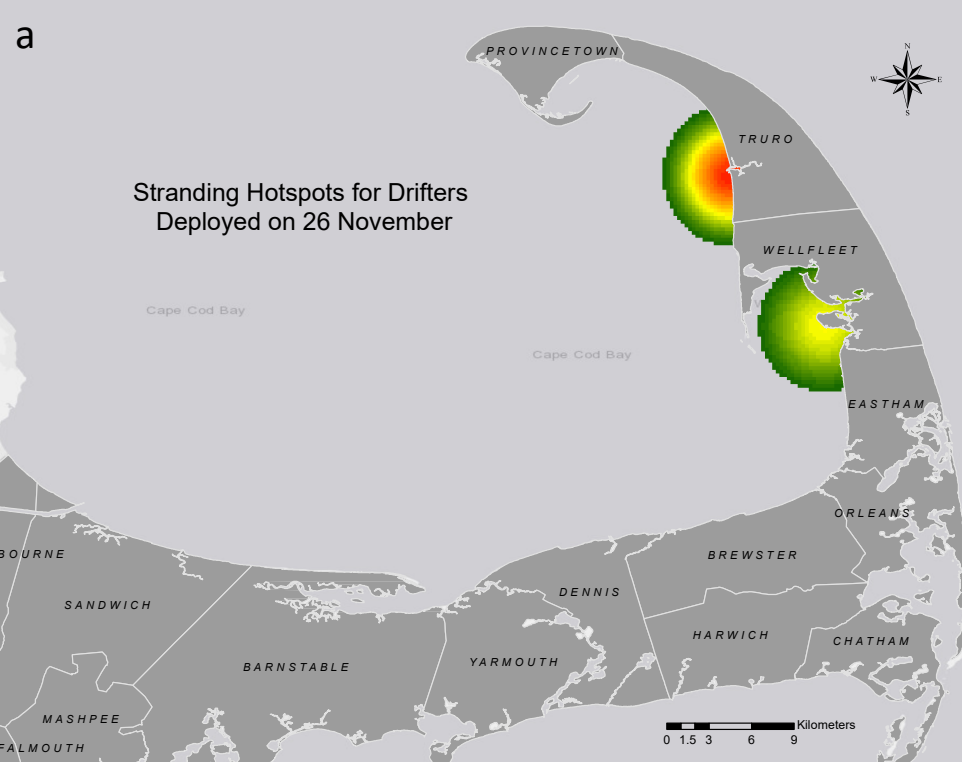

b

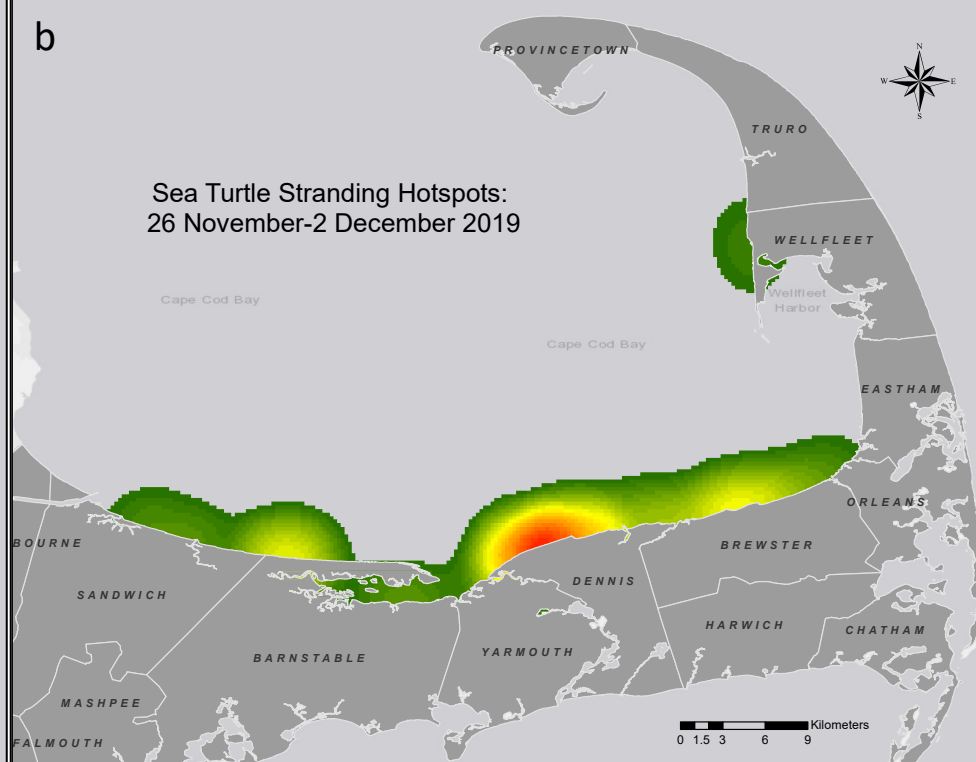

Supplement: Supplemental Information 2 — (a) Drifters (n = 5) deployed on 26 November. (b) Cold-stunned sea turtle strandings (n = 72) from 26 November–02 December. Red indicates the highest number of data points, while yellow indicates intermediate and green indicates the lowest number. [file peerj-11-15866-s002.pdf]
